# Neural response during emotion regulation in monozygotic twins at high familial risk of affective disorders

**DOI:** 10.1016/j.nicl.2018.11.008

**Published:** 2018-11-13

**Authors:** Iselin Meluken, Ninja Meinhard Ottesen, K. Luan Phan, Phillipe R. Goldin, Martina Di Simplicio, Julian Macoveanu, Hartwig Roman Siebner, Lars Vedel Kessing, Maj Vinberg, Kamilla Woznica Miskowiak

**Affiliations:** a Copenhagen Affective Disorder research Centre (CADIC), Psychiatric Centre Copenhagen, University Hospital of Copenhagen, Rigshospitalet, 6233, Blegdamsvej 9, 2100 Copenhagen, Denmark; b Danish Research Centre of Magnetic Resonance, Copenhagen University Hospital, Kettegård Alle 30, 2650 Hvidovre, Denmark; c Department of Psychiatry, University of Illinois at Chicago, 1747 Roosevelt Rd, Chicago, IL 60608, USA; d University of California Davis, 135 Young Hall, One Shields Avenue, CA 95616, USA; e Centre for Psychiatry, Imperial College London, South Kensington Campus, London SW7 2AZ, UK; f Department of Neurology, Copenhagen University Hospital, Bispebjerg, University of Copenhagen, Hvidovre Hospital, Kettegård Alle 30, 2650 Hvidovre, Denmark; g Department of Psychology, University of Copenhagen, Øster Farimagsgade 2A, 1353 København K, Denmark

**Keywords:** Mood disorder, Monozygotic twins, Endophenotypes, Cognitive neuroscience, Magnetic resonance imaging

## Abstract

**Purpose:**

We investigated the neural correlates of emotion regulation and -reactivity in adult unaffected monozygotic twins with a co-twin history of unipolar or bipolar disorder (high-risk), remitted or partially remitted twins with a personal history of unipolar or bipolar disorder (affected) and twins with no personal or first-degree family history of unipolar or bipolar disorder (low-risk).

**Methods:**

We assessed 37 high-risk, 56 affected and 28 low-risk participants. Participants viewed unpleasant and neutral pictures during functional magnetic resonance imaging and were instructed to down-regulate their emotional response through reappraisal or mental imagery, as well as to maintain the elicited emotion.

**Results:**

After adjusting for subsyndromal depressive symptoms, bilateral supplementary motor areas, posterior dorsal anterior cingulate cortices and the left frontal eye field showed less activity during reappraisal of unpleasant pictures in high-risk than low-risk participants. Notably, affected participants did not differ from high-risk or low-risk participants in neural response during reappraisal. There were no group differences in ventrolateral prefrontal cortex seed based functional connectivity during reappraisal or neural response during mental imagery or emotional reactivity.

**Conclusion:**

Lesser response in dorsal midline areas might reflect familial risk related abnormalities during down regulation of emotional reactivity through reappraisal.

## Highlights

• High-risk twins showed less reappraisal related activity than low-risk twins
• The reduced activity in high-risk twins was observed in dorsal midline regions
• This group difference prevailed after adjustment for depressive symptoms

## 1 Introduction

Impaired emotion regulation and heightened emotional reactivity are common features of unipolar (UD) and bipolar disorders (BD) (Hofmann et al., 2012; Mennin et al., 2007; Nolen-Hoeksema et al., 2008). For example, depression is associated with greater use of maladaptive than adaptive emotion regulation strategies (e.g. avoidance, rumination and suppression) (Aldao et al., 2010). Although more self-reported use of maladaptive emotion regulation strategies and abnormal neural response during automatic regulation of negative affect also has been observed in individuals at familial risk of depression (Ian H. Gotlib et al., 2014), it is unclear whether such genetically predisposed individuals display abnormalities in cognitive and neural measures of *voluntary* emotion regulation.

Risk factors have essential translational relevance by increasing our understanding of the underlying mechanisms that precede – or prevent – illness onset. Unaffected monozygotic (MZ) twins from discordant twin pairs provide a unique measure of familial risk, as they have a very high familial risk given their identical genetic make-up to their affected co-twins (Boomsma et al., 2002). Risk factors are best investigated in longitudinal studies due to the continuous risk of disease onset (Weissman et al., 2016). However, cross-sectional comparisons between individuals with affective disorder, individuals with familial risk, and individuals without familial risk enable investigation of whether certain illness related abnormalities meet the endophenotype criterion of trait related phenomena that are present in a higher degree in family members than in the general populations (Gottesman and Gould, 2003). Because unaffected individuals with familial risk by definition have managed to withstand disease onset, they may also display compensatory adaptation and resilience-related factors. Specifically, abnormalities shared by high-risk and affected groups meet the *risk endophenotype* criterion, while phenomena found shared by high -and low-risk unaffected twins may reflect *resilience*, and changes found specifically in high-risk individuals are likely to represent *compensatory adaptation* to familial risk (Wiggins, 2017).

Emotion regulation refers to a variety of strategies used to modulate emotion in a goal directed manner. Reappraisal is a specific method for voluntary emotion regulation that entails reinterpretation or distancing from stimulus-related emotion reactivity (Etkin et al., 2011). Meta-analyses have shown that at a neural level, reappraisal involves a network of regulatory brain regions including dorsolateral prefrontal cortex (dlPFC), ventrolateral PFC (vlPFC), dorsomedial PFC (dmPFC), dorsal anterior cingulate cortex (dACC), supplementary motor area (SMA), pre-SMA and parietal regions that function to down-regulate hyperactivity in emotion generative brain regions (e.g. amygdala) (Buhle et al., 2014; Frank et al., 2014; Kohn et al., 2014). Dysfunction within the reappraisal network is a feature of affective disorders (Picó-Pérez et al., 2017; Zilverstand et al., 2017). Specifically, depressed and euthymic patients with BD or UD display in response to unpleasant pictures reduced prefrontal top-down control of amygdala (Zilverstand et al., 2017) and decreased recruitment of the vlPFC (Picó-Pérez et al., 2017; Zilverstand et al., 2017), a region involved in selection of goal appropriate responses (Ochsner et al., 2012). Only two studies have investigated the neural correlates of reappraisal of unpleasant affective pictures in individuals at familial risk of BD (Kanske et al., 2015) or UD (Simsek et al., 2017). Individuals at familial risk of BD showed deficient down-regulation of amygdala responses and more *positive* functional connectivity (i.e. co-activation over time) between amygdala and orbitofrontal cortex during reappraisal (Kanske et al., 2015). This may reflect unintended emotion amplification given the association between positive amygdala–ventral PFC functional coupling and enhanced negative emotion. In contrast, no aberrant cortico-limbic activity was found in UD at-risk groups (Simsek et al., 2017).

An alternative method for generating and regulating emotions is mental imagery. Mental imagery seems to elicit greater emotional reactions in individuals with mood instability (Di Simplicio et al., 2016) and has been purposed as an ‘emotion amplifier’ (Holmes et al., 2008; Ivins et al., 2014). In keeping with this, imagery-based emotion regulation techniques have been shown to hold potential to reduce mood instability in BD (Holmes et al., 2016). However, no investigation of its neural underpinnings in association with familial risk of affective disorders has been conducted.

## 2 Aims of the study

We investigated functional magnetic resonance blood‑oxygen-level dependent signal during emotion regulation through reappraisal or mental imagery and during emotional reactivity to unpleasant pictures in three distinct groups: (1) unaffected monozygotic twins with a co-twin history of unipolar or bipolar disorder (high-risk), (2) remitted or partially remitted monozygotic twins with a personal history of unipolar or bipolar disorder (affected) and (3) monozygotic twins with no personal or first-degree family history of unipolar or bipolar disorder (low-risk). The primary hypothesis was that prefrontal activity during reappraisal of unpleasant pictures and ventrolateral prefrontal seed based functional connectivity during reappraisal would meet the endophenotype criterion by being aberrant in high-risk and affected participants compared with low-risk participants. We also conducted secondary volume of interest analyses of neural activity for emotion regulation through mental imagery and for emotion reactivity. In addition to these primary and secondary three-way interaction analyses, we conducted explorative pairwise group comparisons and whole brain analyses for hypothesis generating purposes.

## 3 Methods

### 3.1 Participants and recruitment

We used a nationwide linkage of the Danish Twin Registry (Skytthe et al., 2013) and the Danish Psychiatric Central Research Register (Mors et al., 2011) to identify eligible participants. These were monozygotic twins registered with a personal or co-twin history of an affective spectrum diagnosis (ICD-10 codes: F30–34.0 and F38.0) between January 1995 and June 2014, and 18–50 years of age. Age and sex matched participants with low familial risk were identified as not having a personal or co-twin history of an affective spectrum diagnosis. Initially, an invitation letter was sent by post to eligible participants. After approximately two weeks, all invited participants were contacted by phone to seek acceptance of participation and screen for exclusion criteria. If no contact was established, several attempts were made to contact eligible participants by phone and, if necessarily, a second invitation letter was posted. Participants were excluded if their birth weight was under 1.3 kg, if they had current severe somatic illness, a history of brain injury, current substance abuse, current mood episode defined as Hamilton Depression Rating Scale (HDRS-17) or Young Mania Rating Scale (YMRS) scores >14, were pregnant or were found to be dizygotic by pairwise DNA tests. Additionally, participants with low familial risk were excluded if they reported any first-degree relative with organic, schizophrenia spectrum or affective disorders. Participants' use of psychotropic medication did not entail exclusion to increase generalizability of findings. All participants gave informed consent to the study conducted according to the Helsinki declaration. The study was approved by the local ethics committee (H-3-2014-003) and the Danish data protection agency (2014-331-0751).

### 3.2 Procedure and clinical assessment

Participants were invited to attend a one-day assessment from 8:30 a.m. to 5 or 7 p.m. Participants underwent biological data sampling, subjective and objective ratings, a diagnostic interview, neurocognitive testing and lastly functional magnetic resonance imaging (fMRI) scans at the Danish Research Centre for Magnetic Resonance (DRCMR) at Copenhagen University Hospital Hvidovre. Life-time diagnoses of psychiatric illness were assessed using the Schedules for Clinical Assessment in Neuropsychiatry (SCAN) (Wing et al., 1990). All those with a personal or co-twin history of moderate to severe UD (F32.1-33.9) or BD (F31.0-31.9) were grouped as affected or high-risk participants respectively, whereas those without were grouped as low-risk participants. Twins who were identified through the diagnostic interview as affected by, or at risk of, schizoaffective disorder were included. Moreover, twins who were identified with depressive and mixed states were included as affected by BD in line with the ICD-10. In cases where only one twin from a twin pair accepted to participate or one twin met exclusion criteria, single twins were included. Their high- or low-risk status was determined according to the co-twin's diagnoses in the Danish Central Research Register. Observer -and self-rating instruments included the HDRS-17 (Hamilton, 1967), the YMRS (Young et al., 1978), the Major Depression Inventory (MDI) (Bech et al., 2001), the State-Trait Anxiety Inventory form Y (STAI-Y) (Spielberger, 1989) and the Coping Inventory of Stressful Situations (CISS) – Danish version (Endler and Parker, 1990). Premorbid verbal intelligence was estimated with the Danish Adult Reading Task (DART) (Nelson and O'Connell, 1978). Finally, handedness was assessed with the Edinburgh Handedness 10-item Inventory (Oldfield, 1971). All assessors were blinded for participants' risk status.

### 3.3 Assessment of emotion regulation and reactivity

#### 3.3.1 fMRI paradigm

We used a paradigm including blocks of neutral or unpleasant pictures from the International Affective Picture System (IAPS) (Lang et al., 1995) coupled with instructions to down-regulate or to maintain the emotions elicited (see Table S1 for details). Blocks of unpleasant pictures were preceded by an instruction to ‘reappraise’, ‘change image’ or ‘maintain’, while blocks of neutral pictures were preceded by an instruction to ‘just look’. In ‘reappraise’ blocks, participants were instructed to reduce their emotional reactivity to unpleasant pictures by *reinterpreting* the content of the pictures to facilitate a less negative emotional response. An example of reinterpretation is A picture of people crying outside a church was in fact a picture of people experiencing tears of joy after a wedding’. For the ‘change image’ blocks, participants were instructed to modify the visual properties of the pictures by using mental imagery to facilitate a less negative emotional response. For example: ‘Imagine the pictures including diagonal stripes or a funny object’. In the ‘maintain’ blocks, participants were instructed to hold on to the emotional state elicited by the unpleasant pictures. Finally, in neutral ‘just look’ blocks, participants were instructed to view pictures as they normally would. Prior to fMRI scanning, participants practiced executing the ‘reappraise’ and ‘change image’ instructions and gave examples of how they reinterpreted or changed images. This ensured that they understood these instructions correctly. After each block of four pictures, participants rated their subjective negative emotional responses on a scale from 1 to 4 (1 = no unpleasantness; 4 = greatest unpleasantness) by button pressing. Participants' ratings and response times were recorded. Eight trials of each block type were presented interleaved by a 12 s baseline block of a fixation cross on a black screen, leading to a task time of 19 min. All participants performed the task in one single run. Each block consisted of an instruction (4 s), four pictures (4 s per picture) balanced for valence and arousal without an inter-stimulus interval and rating (4 s). After the scan, participants were asked whether they felt they had managed to apply the reappraisal and change image strategies and to give specific examples.

#### 3.3.2 Behavioural assessment outside the scanner

Emotion regulation and reactivity in prototypical positive and negative social scenarios were assessed with a novel in-house computerized task (Goldin et al., 2009). Participants were instructed to either ‘dampen’ or ‘maintain’ their positive and negative emotions elicited by the scenarios. In ‘dampen’ blocks, participants were instructed to downregulate their emotional response without specific training other than an example of how to dampen through reappraisal. In ‘maintain’ blocks, participants were instructed to react naturally and maintain the emotion elicited by the scenarios. For the ‘dampen’ and ‘maintain’ conditions, the paradigm consisted of two positive and two negative social scenarios, as well as one neutral scenario. All nine scenarios were presented as 11 short written paragraphs about a particular social scenario including 10 associated positive or negative self-belief statements (e.g. ‘you are outstanding’ or ‘you don't fit in’). All self-belief statements were followed by a rating of emotional state on a visual analogue scale from 1 to 100 representing degree of discomfort/sadness or pleasure/happiness.

### 3.4 fMRI data acquisition

All fMRI scans were acquired using a 3 Tesla Siemens Verio scanner and a 32-channel head array receive coil. A total of 502 volumes of T_2_*-weighted echo planar imaging (EPI) images were acquired with parallel imaging (GRAPPA) and whole-brain field of view (acceleration factor = 2; FOV = 192 mm^2^; matrix size = 64 × 64; imaging plane = axial; slice thickness = 3 mm; no. of slices = 42; acquisition order = interleaved upwards; echo time = 30 ms; repetition time = 2320 ms; flip angle = 80°). T1-weighted images were acquired for subject alignment, using an MPRAGE sequence (FOV = 230 mm^2^; ST = 1.9 mm; no. of slices = 224; slice thickness = 1 mm; repetition time = 1900 ms; echo time = 2320 ms; flip angle = 9°). Participants' pulse and respiration were recorded during scanning.

### 3.5 Analysis of fMRI data

#### 3.5.1 Pre-processing and first-level analysis

Data pre-processing and first-level analysis were conducted using FEAT version 5.0.9, part of the FMRIB Software Library (“FSL – FslWiki,” n.d.). Pre-processing included non-brain removal, linear and nonlinear registration to structural space, normalization to the Montreal Neurological Institute (MNI) standard space, motion correction, spatial smoothing (full width half maximum = 5 mm) and grand mean intensity normalization of the 4D datasets. Correction for geometric EPI distortions was performed based on an acquired B_0_ field map. All participants' registration and unwarping results were visually controlled. Additionally, before high pass temporal filtering (cut-off = 307 s.), ICA-AROMA, an independent component analysis (ICA) based strategy for automatic removal of motion artefacts was carried out (Pruim et al., 2015). Finally, ICA-based denoising was manually performed using MELODIC (Beckmann and Smith, 2004) to remove components resulting from acquisition artefacts.

The first-level general linear model included four task regressors of interest modelling response to unpleasant ‘reappraise’, ‘change image’ and ‘maintain’ picture blocks and to neutral pictures. We included a regressor of no interest modelling emotion ratings and a regressors modelling out blocks where participants failed to rate their degree of unpleasantness, if the number of missed ratings exceeded two standard deviations of the mean. All regressors were convolved with a double-gamma haemodynamic response function. In addition, temporal derivatives of task regressors were included to model slice-timing effects. Physiological noise modelling was performed for cardiac and respiratory noise, creating 16 additional regressors (Brooks et al., 2008). A priori contrasts of interests were emotion regulation through reappraisal (reappraise > maintain), emotion regulation through mental imagery (change image > maintain) and emotion reactivity (maintain > just look).

#### 3.5.2 Group level analysis

For the primary and secondary analyses, we compared high-risk, affected and low-risk participants with an F-test. Significant and trend level F-tests were followed by post-hoc pairwise group comparisons. In explorative analyses we compared the three groups with pairwise *t*-tests.

Group-level analyses were conducted using permutation inference with Permutation Analysis of Linear Models (PALM) (Winkler et al., 2014). Dependence within twin pairs was modelled by restricting permutation to within and between twin pairs and between single twins (Winkler et al., 2015). Participants' contrasts images were fed into separate ANOVAs and all group comparisons of affected, high-risk and low-risk participants were performed with and without adjustment for subsyndromal depressive symptoms (i.e. HDRS-17 scores). The main effects of task were examined with a one-sample *t*-test across all participants unadjusted for subsyndromal depressive symptoms. Analyses were conducted within structurally based regions of interest (ROI) and exploratory across the whole brain. For the emotion regulation contrasts (i.e. reappraisal and mental imagery), we used a mask of PFC including activation peaks from a recent meta-analysis of reappraisal of emotional stimuli in affective disorders (Picó-Pérez et al., 2017). Specifically for the mental imagery condition, we also used a mask including areas previously found to be activated during mental imagery (Knauff et al., 2000). We also used masks of left and right amygdala given the amygdala involvement in reactivity to aversive pictures (Costafreda et al., 2008) and aberrant activity during emotion processing in affective disorders (Chen et al., 2011; Palmer et al., 2015). See supplementary material for details of ROI masks.

Statistical inference was based on cluster-wise thresholding with the threshold-free cluster enhancement method (TFCE) (Smith and Nichols, 2009) and Family-Wise Error (FWE) corrected *P*-values < 0.05. Peak activations were reported in MNI coordinates. Additionally, cerebral regions and Brodmann areas were identified through Talairach conversion of the MNI coordinates using GingerALE (“brainmap.org | GingerALE,” 2017) and the Talairach and Tournoux anatomical atlas (Talairach & Tournoux, 1988).

Mean percent blood-oxygen-level dependent (BOLD) signal change was extracted from clusters showing between group differences with the featquery tool for illustrative purposes and post-hoc correlation analysis. We investigated the correlations between BOLD signal and subsyndromal depressive symptoms, emotion ratings during fMRI and to social scenarios outside the scanner (reappraisal: ratings during reappraisal subtracted from ratings during maintain; reactivity: ratings during maintain subtracted from the neutral conditions).

#### 3.5.3 Functional connectivity analysis

Psychophysiological interaction (PPI) analyses were conducted to examine functional connectivity for the reappraise > maintain contrast. We used the left and right vlPFC as seed regions because of the central role of vlPFC in reappraisal and reinterpretation specifically (Kohn et al., 2014; Ochsner et al., 2012; Picó-Pérez et al., 2017; Wager et al., 2008). See supplementary material for details. The seed regions' time-course was entered in a PPI model including all original regressors and four additional PPI interaction regressors (just look × time series, maintain × time series, reappraise × time series, and change image × time series). The left and right vlPFC were used as seed regions in two separate group-level PPI analyses of the reappraise > maintain contrast across the whole brain.

#### 3.5.4 Analysis of behavioural data

Behavioural data were examined with mixed model analysis of variance with group (high-risk, affected, low-risk) as fixed factor and twin pairs as random factors. Moreover, emotion regulation and reactivity were analysed with repeated measurements models using instructions and valence of stimuli as levels and additionally subjects as random factor. Specifically, emotion regulation to unpleasant IAPS pictures were analysed as reappraise or change image vs. maintain, and to negative or positive social scenarios as dampen vs. maintain instructions. Emotion reactivity to IAPS pictures were analysed as maintain vs. neutral and to social scenarios as maintain positive or negative vs. neutral stimuli. Data analyses were conducted in SAS 9.4 (SAS Institute Inc.).

## 4 Results

### 4.1 Participants

From the original 204 participants with full behavioural, clinical and biological data sets, we included 134 participants in the fMRI part of the study in a consecutive manner. Reasons for not scanning 70 participants were either that the target of a minimum of 120 successful fMRI scans was reached (*n* = 16), participants' declination (*n* = 16), metal in body or head trauma (*n* = 4) or other reasons (*n* = 5). Additionally, we refrained from scanning some affected participants with UD to obtain a more balanced sample (*n* = 29). Six participants did not complete the scan session due to claustrophobia, nausea or pain, and two because of technical issues. Of the 126 participants who successfully underwent the emotion regulation and reactivity paradigm, four participants were excluded from analysis because their number of missing ratings exceeded three standard deviations of the mean and one due to a technical issue. Analysis of neural response during emotion regulation and reactivity to IAPS pictures included 121 participants (high-risk: *n* = 37; affected: *n* = 56; low-risk: *n* = 28).

Demographic and clinical characteristics are presented in Table 1. The sample included 10 concordant (BD/BD: *n* = 2; UD/UD: *n* = 4; BD/UD: *n* = 4), 20 discordant (high-risk/UD: *n* = 13; high-risk/BD: *n* = 7), 11 low-risk complete twin pairs and 39 single twins. The three groups were well balanced with respect to age, sex, education and premorbid IQ (*P*s ≥ 0.28), but there were more left-handed participants in affected and high-risk groups than in the low-risk group (*P* = 0.03). As expected, the affected participants in full (HDRS-17 ≤ 7: *n* = 48) or partial remission (HDRS-17 12–8: *n* = 8) scored higher on depressive and anxiety symptoms compared with high-risk and low-risk participants (*P*s ≤ 0.01). Affected participants reported more use of emotion-oriented coping than high-risk and low-risk participants (*P* = 0.002), while there was a trend of high-risk participants reporting more use of task-oriented coping than affected participants (*P* = 0.07), consistent with the results from the entire twin cohort (personal communication, 49).

**Table 1:** Demographic and clinical comparison of affected, high-risk and low-risk MZ twins (*n* = 121).

	Affected (*n* = 56)	High-risk (*n* = 37)	Low-risk (*n* = 28)	*P*	
*Demographic and clinical*^a^, ^d^					
Age, mean (range), years	37 (20–52)	36 (19–52)	38 (19–52)	0.48	
Sex, %, women	70	68	71	0.69	
Education, mean, years	15.0 (14.1–15.8)	15.7 (14.6–16.7)	15.7 (14.5–16.9)	0.48	
Premorbid IQ^b^, mean	113.8 (112.1–115.5)	111.9 (109.7–114.2)	111.0 (106.1–115.9)	0.28	
Left handedness, %, LQ < 0	20	22	4	0.03	AF&HR > LR
Bipolar/HR bipolar, %	38	27	NA		
Unipolar/HR Unipolar, %	62	73	NA		

*Medication, yes*					
Any psychotropic, %	57	5	0		
SSRI, SNRI or TCA, %	43	3	0		
Antipsychotic, %	20	0	0		
Mood stabilizer^c^, %	25	0	0		
Benzodiazepine, %	7	3	0		
Methylphenidate, %	2	0	0		

*Symptoms*					
HDRS-17, mean	4.4 (3.7–5.1)	2.6 (1.7–3.5)	1.9 (0.9–2.9)	<0.001	AF > HR&LR
YMRS, mean	2.0 (1.5–2.5)	1.6 (1.0–2.2)	1.3 (0.6–2.0)	0.21	
MDI, mean (range)	10.4 (31.0–0.0)	5.8 (26.0–0.0)	3.3 (16.0–0.0)	0.001	AF > HR&LR
State anxiety, mean	30.2 (28.5–32.1)	27.4 (25.4–29.5)	26.1 (24.0–28.5)	0.01	AF > HR&LR
Trait anxiety, mean	39.9 (38.1–41.9)	33.1 (31.3–35.1)	33.6 (31.1–36.3)	<0.001	AF > HR&LR

*Habitual coping strategies*					
Task-oriented, mean	52.4 (49.6–55.4)	57.3 (54.1–60.5)	54.7 (51.1–58.3)	0.07	AF < HR
Emotion-oriented, mean	43.5 (40.2–46.9)	35.4 (31.5–39.3)	33.2 (26.1–40.3)	0.002	AF > HR&LR
Avoidance-oriented, mean	40.4 (38.1–42.6)	38.0 (36.0–40.5)	39.0 (36.0–41.9)	0.36	

Abbreviations: MZ = Monozygotic twins, AF = Affected twins, HR = High-Risk, LR = Low-risk, LQ = Lateral Quotient, NA = Not Applicable, SSRI=Selective Serotonin Reuptake inhibitor, SNRI=Serotonin and Norepinephrine Reuptake Inhibitor, TCA = Tricyclic Antidepressant, HDRS-17 = Hamilton Depression Rating Scale, YMRS=Young Mania Rating Scale, MDI = Major Depression Inventory.

a Data are presented as estimated means accounting for intra twin pair-dependence

b Eight participants with dyslexia were excluded

c Lithium, anticonvulsants

d Benzodiazepine, methylphenidate

Table 1. Descriptive variables are presented as estimated group means with confidence intervals (in brackets) by a mixed model procedure accounting for within twin-pair dependence. Group comparisons of monozygotic twins with affective disorder (affected), at risk of affective disorder (high-risk) or at low-risk of affective disorder (low-risk) are reported with *p*-values and as are results from post-hoc pairwise group comparisons.

### 4.2 Functional magnetic resonance imaging results

Results are presented in Table 2. Because one high-risk twin reported inability to use reappraisal as downregulation strategy, analyses of this condition included 120 participants.

**Table 2:** Main effects of task and group comparisons of affected, high-risk and low-risk MZ twins of emotion regulation and reactivity during fMRI.

Search area	Region	BA	MNI x y z			Voxels	Peak p-value
Maintain > just look							
*Main effects across all participants*							
Whole brain	Lingual gyrus	18	10	−84	0	215	0.03

Reappraise > maintain							
*Main effects across all participants*							
PFC	Middle frontal gyrus	46	−40	34	10	6732	<0.001
	Inferior frontal gyrus	44	−46	8	20	–	
	Middle frontal gyrus	46	46	34	12	4283	<0.001
	Inferior frontal gyrus	44	48	12	32	–	
	Superior frontal gyrus	10	−22	50	12	34	0.045
Whole brain	Superior Parietal Lobule	7	−26	−58	54	6449	<0.001
	Superior Parietal Lobule	7	22	−74	60	5044	0.001
	Superior frontal gyrus	8	−2	28	44	353	0.03
	Middle frontal gyrus	6	−30	6	64	312	0.02
	Middle frontal gyrus	46	−40	32	12	2590	0.001
	Inferior frontal gyrus	44	−46	8	24	–	
	Middle frontal gyrus	46	46	36	12	2120	0.005
	Middle frontal gyrus	9	50	12	34	–	
	Inferior frontal gyrus	47	−34	38	−12	34	0.03
	Anterior Insula	13	−28	22	−2	293	0.03
*High-risk* vs. *affected* vs. *low-risk*							
PFC	Medial frontal gyrus (SMA)	6	−2	−6	50	2	0.049
PFC _HDRS_^a^	Medial frontal gyrus (SMA)	6	−2	−6	50	6	0.048
*High-risk < low-risk*							
PFC	Medial frontal gyrus (SMA)	6	−2	−6	50	589	0.03
PFC _HDRS_^a^	Medial frontal gyrus (SMA)	6	−2	−6	50	819	0.02
	Superior frontal gyrus (FEF)	6	−20	0	56	26	0.04
*High-risk* vs. *affected* vs. *low-risk*							
Whole brain _HDRS_^a^	Medial frontal gyrus (SMA)	6	−2	−6	50	16	0.09
*High-risk < low-risk*							
Whole brain _HDRS_^a^	Medial frontal gyrus (SMA)	6	−2	−6	50	677	0.03
	Postcentral Gyrus	3	−60	−20	38	79	0.03
	Superior frontal gyrus (FEF)	6	−20	0	54	21	0.046

*Explorative*^b^*high-risk < low-risk*							
Whole brain	Medial frontal gyrus (SMA)	6	−2	−6	50	75	0.04
	Cingulate gyrus	32	8	6	46	39	0.045
	Cingulate gyrus	24	−8	14	34	18	0.045

Change image > maintain							
Explorative^b^ affected < low-risk							
Imagery	Precuneus	7	2	−68	38	311	0.07

- Refers to local maxima within cluster.

Abbreviations: fMRI = Functional Magnetic Resonance Imaging, ROI = Region of Interest, FWE = Family Wise Error, BA = Brodmann Area, MNI, = Montreal Neurological Institute, PFC = Prefrontal Cortex, SMA = Supplementary Motor Area, HDRS = Hamilton Depression Rating Scale, FEF = Frontal Eye Field.

a HDRS refers to adjustment for subsyndromal depressive symptoms.

b Pairwise tests are reported as explorative when *P* value from the F-test is >0.1.

#### 4.2.1 Main effects of task across participants

Reappraisal (reappraisal > maintain) activated bilateral middle and inferior frontal gyri (MiFG, IFG) and left superior frontal gyrus (SFG) within the PFC. However, there were no main effects of reappraisal in functional connectivity analyses from the left or right vlPFC or in amygdala ROI analyses. Since we found no main effect of emotion regulation on functional connectivity from the vlPFC to other brain regions, we decided to not go ahead with the planned group comparisons. There were no main effects of downregulation through mental imagery (change image > maintain) within the PFC, the mental imagery ROI or within left or right amygdala. Finally, there were no main effects of emotion reactivity (maintain > just look) within left or right amygdala.

In the exploratory whole brain analysis, reappraisal activated bilateral superior parietal lobuli and MiFG and left IFG, anterior insula and medial SFG. Mental imagery did not produce main effects in the whole brain analysis, whereas emotion reactivity revealed increased activity in the right lingual gyrus.

#### 4.2.2 Primary group comparisons of emotion regulation through reappraisal

**Fig. 1:**
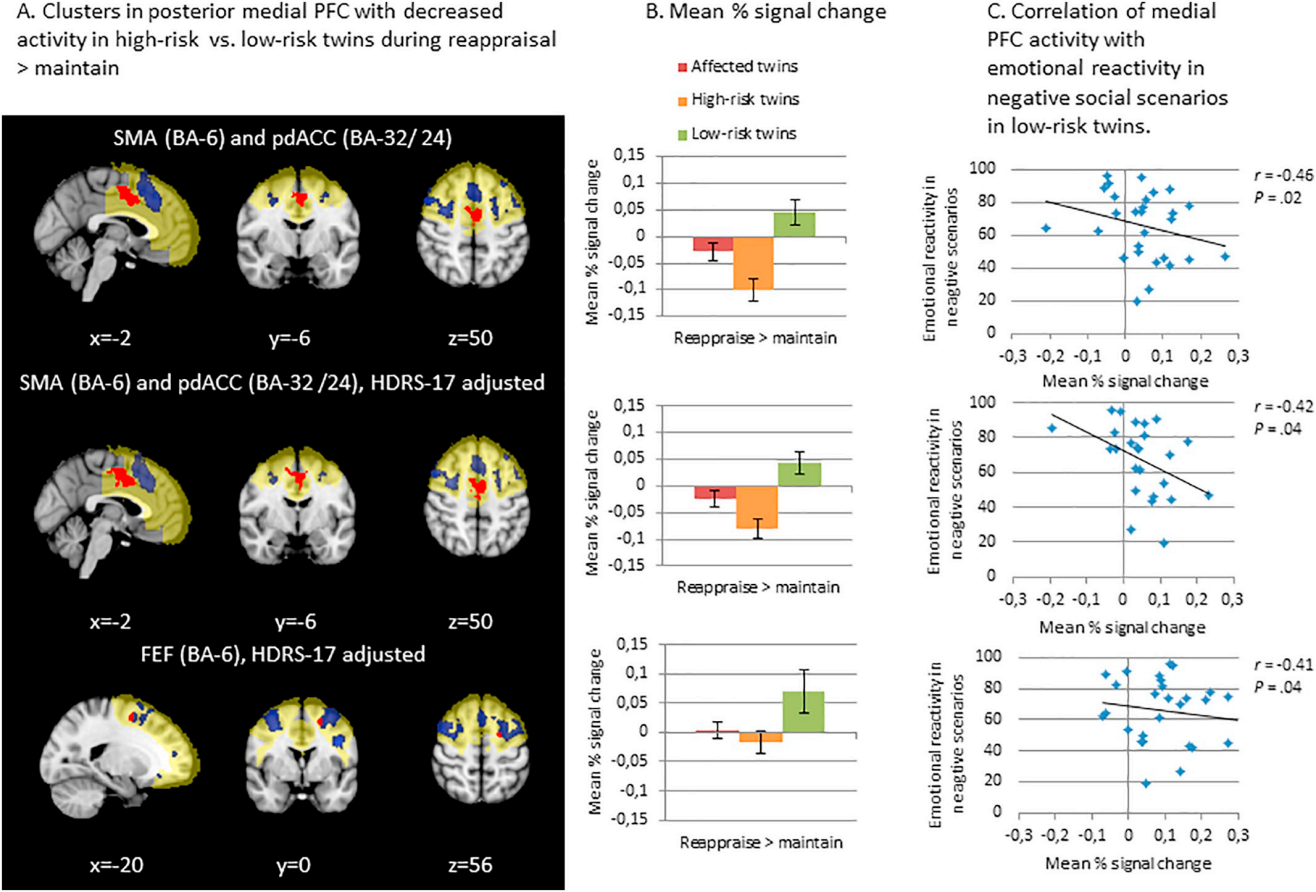
Panel A displays decreased activity in posterior medial PFC during reappraisal in monozygotic (MZ) twins at high-risk of affective disorders compared with low-risk MZ twins. When adjusted for subsyndromal depressive symptoms, these high-risk MZ twins displayed decreased activity in bilateral SMA and pdACC and the left FEF compared with low-risk MZ twins. The Region of interest (ROI) is marked in yellow, the main effect of task within the ROI across participants is marked in blue and the group difference between high – and low-risk participants is marked in red. Panel B displays the mean percent signal change within the corresponding same-row significant groupwise differences (clusters in red) from Panel A in affected, highrisk and low-risk participants. Panel C displays that low-risk participants' decreased activity in SMA, pdACC and the FEF during reappraisal correlated with more negative emotional reactivity to negative social scenarios. Across panel A, B and C the first row displays results from analyses without subsyndromal depression scores as covariate (i.e. HRDS-17 scores), while rows two and three display results from analyses with subsyndromal depression scores as covariate. Error bars represent standard error of the mean, asterisks represent statistically significant group differences, r = correlation coefficient. PFC = Prefrontal cortex, HDRS-17 = Hamilton Depression Rating Scale-17, BA = Brodmann Area, SMA = Supplementary Motor Area, FEF = Frontal Eye Field, pdACC = posterior dorsal anterior cingulate cortex.

The F-test of BOLD signal for reappraisal (reappraisal > maintain) revealed a difference across high-risk, affected and low-risk groups in the SMA (BA-6) in left medial frontal gyrus (MeFG) both with and without adjustment for subsyndromal depressive symptoms within the PFC. Post-hoc pairwise comparisons revealed that this difference was driven by lower activity in high-risk than low-risk participants bilaterally in the SMA (BA-6) extending into the pdACC (BA-24 and BA-32) and additionally in the left FEF (BA-6) in the SFG when adjusting for subsyndromal depressive symptoms (Fig. 1). There were no other differences in post-hoc pairwise group comparisons.

In the brain-behaviour correlation analysis, low-risk participants displayed correlation between less activity in SMA, pdACC (*r* = −0.45 and − 0.42) and the left FEF (*r* = −0.41) during reappraisal and more negative emotional reactivity in negative social scenarios outside the scanner (Fig. 1). Neither affected nor high-risk participants displayed such correlation (*P*s ≥ 0.42). There was no correlation between BOLD signal during reappraisal and subsyndromal depressive symptoms in any of the three groups (*Ps* ≥ 0.22).

#### 4.2.3 Secondary group comparisons of emotion regulation through mental imagery and emotion reactivity

There were no group differences, with or without adjustment for subsyndromal depressive symptoms, in activations within the imagery ROI during emotion regulation through mental imagery (change image > maintain) or within amygdala during emotion reactivity (maintain > just look).

#### 4.2.4 Explorative analyses: whole-brain activations and pairwise comparisons

During reappraisal there was a trend towards a difference in neural response between the three groups across the whole brain that was driven by lesser activity in the SMA, the left FEF and left postcentral gyrus in high-risk vs. low-risk participants (see supplementary Fig. 3). Further, exploratory pairwise comparisons during reappraisal across the whole brain unadjusted for depressive symptoms revealed lower activity in left MeFG and bilateral ACC in high-risk vs. low-risk participants.

Pairwise comparisons of emotion regulation through mental imagery revealed a trend of affected participants displaying lower activity than the low-risk participants in the precuneus within the imagery ROI unadjusted for depressive symptoms (see supplementary Fig. 4).

### 4.3 Behavioural results

Ratings during fMRI were analysed for 118 participants (high-risk: *n* = 36; affected: *n* = 54; low-risk: *n* = 28) because of response box failure during scanning of three participants. Ratings during the pre-fMRI social scenarios task were analysed for 101 participants (high-risk: *n* = 28; affected: *n* = 49; low-risk: *n* = 24) because eight participants were excluded due to dyslexia and data from 12 participants were lost due to computer problems. Ratings and response times are displayed in supplementary Figs. 5 and 6.

#### 4.3.1 Emotion regulation

Participants were able to downregulate their negative emotional responses to unpleasant pictures through reappraisal and mental imagery, as indicated by ratings of lesser negative emotion in these downregulation vs. maintain conditions (‘reappraise’: *F* = 57.2, df = 1, 117, *P* ≤ 0.001; ‘change image’: *F* = 79.0, df = 1, 117, *P* ≤ 0.001). Participants' speed of indicating their emotional reactions was generally longer after downregulation than maintenance of emotions (reappraise: *F* = 17.5, df = 1, 117, *P* < 0.001; change image: *F* = 20.9, df = 1, 117, *P* ≤ 0.001). There was a trend towards an overall effect of group on ratings across reappraise and maintain conditions (*P* = 0.10) that was driven by less negative reactions in high-risk vs. low-risk participants (*P* = 0.03). There were no other overall group differences (*P*s ≥ 0.22) or group by condition interactions (*P*s ≥ 0.43) in ratings or response time to unpleasant pictures during fMRI.

In the social scenarios task outside the scanner, participants were also able to down-regulate their emotions as reflected by ratings of less unpleasant and pleasant emotions during dampen than maintenance conditions (negative: *F* = 147.6, df = 1, 100, *P* ≤.001; positive: *F* = 118.2, df = 1, 99, *P* ≤.001). There were no overall group differences (*P*s ≥ 0.36) or group by condition interactions (*P*s ≥ 0.64) in emotion ratings in response to social scenarios.

#### 4.3.2 Emotion reactivity

During fMRI, unpleasant pictures elicited more negative emotions than neutral pictures, as expected (*F* = 803.9, df = 2, 117, *P* ≤.001). Participants were generally slower to rate their emotional reactions to unpleasant than neutral pictures (*F* = 28.4, df = 1, 117, *P* < 0.001). There was a trend towards a main effect of group on response times (*P* = 0.07), driven by slowed responses in affected vs. low-risk participants (*P* = 0.02). However, there were no main effect of group in emotion ratings (*Ps* ≥ 0.31) or interaction between group and condition for emotion ratings or response times (*P*s ≥ 0.10).

In the social scenarios task, participants rated negative and positive scenarios as eliciting more emotions than the neutral scenario as expected (negative: *F* = 626.3, df = 1, 100, *P* < 0.001; positive: *F* = 810.0, df = 1, 99, *P* < 0.001). There were trends towards interaction between group and valence in social scenarios (negative: *P* = 0.10; positive: *P* = 0.06). This was driven by more negative emotion ratings in affected than low-risk participants during negative scenarios and during the neutral scenario (negative: *P* = 0.02; positive: *P* = 0.99; neutral: *P* = 0.01).

Table 2. Main effects of task and group comparisons are presented by peak cluster localization in Montreal Neurological Institute (MNI) standard space coordinates and cerebral region with corresponding Brodmann area after conversion to Talairach space, cluster size (number of voxels) and peak *P*-values. Results are derived from permutation methods allowing modelling the dependence structure of twin pairs. To define clusters, the threshold free cluster enhancement method was used. Significant results were found by thresholding family-wise corrected images at *P* = 0.05 and trend level results were found by thresholding family-wise corrected images at *P* = 0.10. Significant and trend level results from volumes of interest used as small volume correction and across whole brain are presented, as well are results from analysis unadjusted and adjusted for current subsyndromal depressive symptoms.

## 5 Discussion

In this large (*n* = 121) high-risk MZ twin fMRI study, we primarily investigated PFC activity and vlPFC functional connectivity during downregulation of emotional response to unpleasant pictures through reappraisal in high-risk, affected and low-risk participants. We observed a difference in neural activity between the three groups during reappraisal that was driven by lesser bilateral SMA and pdACC activity in high-risk vs. low-risk participants. When adjusting for subsyndromal depressive symptoms, lesser activity in high-risk participants was also observed in the left FEF. In low-risk participants specifically, lesser fMRI BOLD signal to unpleasant pictures during reappraisal correlated with higher negative emotional reactivity in the pre-fMRI social scenarios task. In addition, during reappraisal, exploratory whole-brain analyses adjusted for subsyndromal depressive symptoms revealed lesser activity in similar midline areas including left parietal cortex in high-risk vs. low-risk participants. Since there were no main effects of emotion regulation in the vlPFC seed based functional connectivit analysis, we decided to not conduct the planned group comparisons of fronto-limbic functional connectivity. We found no differences between groups in secondary investigation of neural response during emotion regulation through mental imagery or emotion reactivity or in the behavioural tasks.

The reappraise > maintain contrast showed a main effect in key areas of the regulatory brain network including bilateral dlPFC, dmPFC and pre-SMA implicating that participants did engage in reappraisal as instructed. The lesser activity during reappraisal in high-risk vs. low-risk participants in bilateral SMA and pdACC and left FEF (Fig. 1) within the PFC seemed robust as a similar group difference was found in exploratory whole-brain analysis and when depression scores were used as covariate for all participants. In fact, regressing out depressive symptoms provides stronger and more direct evidence that the lesser activity in these areas is a risk correlate and not a consequence of depressive symptoms. These group differences contrast with two studies of neural response during reappraisal of emotional pictures in individuals at familial risk of BD (Kanske et al., 2015) and UD (Simsek et al., 2017) that found no aberrant PFC activity. The discrepancy may relate to the previous studies assessing only female participants (Simsek et al., 2017), applying a region of interest not including medial PFC areas (Simsek et al., 2017), covarying for age (Simsek et al., 2017) or averaging responses across negative and positive stimuli (Kanske et al., 2015). Nonetheless, the absence of risk-associated neural differences during simple *reactivity to* emotional pictures is in accordance with these studies (Kanske et al., 2015; Simsek et al., 2017).

The SMA is a primarily motor area (Picard and Strick, 1996) that has been found together with pre-SMA to robustly activate during reappraisal tasks in recent meta-analyses (Buhle et al., 2014; Frank et al., 2014; Kohn et al., 2014). The activation of SMA may relate to mental representation of alternative scenes during the execution of reappraisal (Kohn et al., 2014), an interpretation in line with activation of the SMA during imagined movement (Picard and Strick, 1996). The pdACC has a putative integrative role in voluntary emotion regulation (Kohn et al., 2014) through its anatomical connections with dlPFC and premotor areas (Etkin et al., 2011). Specifically, it is involved in emotional response expression such as autonomic nervous system activity (Etkin et al., 2011) and through the rostral cingulate motor areas in emotion related behaviour selection and generation (Etkin et al., 2011; Kohn et al., 2014; Picard and Strick, 1996). Additionally, both the SMA and pdACC have been found to activate during interoceptive awareness and appraisal (Critchley et al., 2004; Etkin et al., 2011). The FEF is also often activated during reappraisal (Frank et al., 2014) and has been implicated in memory search and cognitive planning (Makino et al., 2004). Consequently, the reduced activity in these areas in high-risk participants may reflect subtle impairments in reappraisal abilities, not identified in behavioural tasks. The correlation between lesser SMA, pdACC and left FEF activity during reappraisal and more negative emotional reactivity in the social scenarios test in low-risk participants, points to a potential regulatory role of these areas in this group specifically. However, in line with activation of the FEF during saccade eye movements (Paus, 1996), it has been purposed that recruitment of motor planning regions such as the SMA and FEF may relate to eye movements during reappraisal tasks (Frank et al., 2014). In fact, it has been shown that reappraisal of pictures involve more shifts over longer distances in ocular fixation than when instructed to maintain emotions (Van Reekum and van Reekum, 2007). Thus, the reduced activity in high-risk versus low-risk participants may also relate to less use of attentional and oculomotor processes activated during reappraisal.

We differences between affected and low-risk participants and thus did not find support of our primary hypotheses that aberrant neural response during reappraisal would show similar abnormalities in affected and high-risk compared with low-risk participants. One explanation could be that psychotropic medication normalized a potentially disrupted response in affected participants (Del-Ben et al., 2012; Godlewska et al., 2016; Hafeman et al., 2014; Posner et al., 2011). Challenging this possibility was no differences in mean percent BOLD signal during reappraisal between medicated and non-medicated participants (*P*s ≥ 0.37). Moreover, post-hoc analysis of all non-medicated participants (*n* = 86) did not reveal differences between affected and low-risk participants during reappraisal (see TableS2 for details). Finally, the absence of main effects of the imagery condition (change image > maintain) suggests that the cognitive task of imagining pictures in a different way did not change neural reactivity to unpleasant pictures in comparison with simply looking at the pictures. Alternatively, the absence of differences could also reflect that imagery-based techniques may be easier to employ compared to standard reappraisal given the similar emotion ratings following these two emotion regulation strategies (see supplementary Fig. 5).

Key strengths of this fMRI study are the large sample of MZ twins, the register-based recruitment and the blinding of assessors to participants' risk status. Nonetheless, several limitations are present in addition to limitations inherent to the cross-sectional design. First, the task time of 19 min increased risk of attenuation of the BOLD response because of possible disengagement from the task. Notably, when comparing behavioural performance in the first vs. last part with post-hoc paired comparisons, we found that participants rated unpleasant pictures as eliciting less negative emotions during maintain (*P* < 0.001) and more during reappraisal (*P* < 0.001) in the last part. This could suggest a degree of attrition across all participants in the latter part of the test. Second, the modest size of the low-risk group (*n* = 29) may have reduced the statistical power. In fact, the “real” sample size was further reduced in statistical analysis because of dependant observation within the 11 complete twin pairs. Third, the PPI analyses from vlPFC during reappraisal did not show any main effects. This was probably because PPI analyses are in general noisy and that the study may therefore have been underpowered for such analyses. Fourth, correlations between BOLD signal in the identified clusters and ‘outside scanner’ behavioural measures are limited by being indirect. However, the rationale for these analyses was that the social scenarios task has shown greater sensitivity to *behavioural measures* of emotion regulation difficulties than reappraisal of affective IAPS pictures (which tend to vary very little across affective and healthy groups, which is suboptimal for correlation analyses) (Kjaerstad et al., 2016). Finally, lack of information on whether affected participants had received psychotherapy involving training of reappraisal techniques, leaves open the question of whether such treatment might have contributed to similar brain activity during reappraisal in the affected and low-risk group unanswered.

The present finding links reappraisal with decreased SMA, pdACC and left FEF activation in monozygotic twins at high familial risk of affective disorders. This reduced activity may reflect neural correlates of high-risk twins' subtle impairments in performing reappraisal despite no behavioural difficulties. This finding warrants prospective studies of reappraisal in high-risk samples to elucidate whether reduced medial PFC activity predicts later disease occurrence.

## Appendix A Supplementary data

Supplementary material Image 1

## References

[bb0005] Aldao A., Nolen-Hoeksema S., Schweizer S. (2010). Emotion-regulation strategies across psychopathology: a meta-analytic review. Clin. Psychol. Rev..

[bb0015] Bech P., Rasmussen N.A., Olsen L.R., Noerholm V., Abildgaard W. (2001). The sensitivity and specificity of the Major Depression Inventory, using the present State Examination as the index of diagnostic validity. J. Affect. Disord..

[bb0020] Beckmann C.F., Smith S.M. (2004). Probabilistic independent component analysis for functional magnetic resonance imaging. IEEE Trans. Med. Imaging.

[bb0025] Boomsma D., Busjahn A., Peltonen L. (2002). Classical twin studies and beyond. Nat. Rev. Genet..

[bb0030] brainmap.org | GingerALE [WWW Document], 2017. http://brainmap.org/ale/. URL http://brainmap.org/ale/ (accessed 11.9.17).

[bb0035] Brooks J.C.W., Beckmann C.F., Miller K.L., Wise R.G., Porro C.A., Tracey I., Jenkinson M. (2008). Physiological noise modelling for spinal functional magnetic resonance imaging studies. NeuroImage.

[bb0040] Buhle J.T., Silvers J.A., Wager T.D., Lopez R., Onyemekwu C., Kober H., Weber J., Ochsner K.N. (2014). Cognitive reappraisal of emotion: a meta-analysis of human neuroimaging studies. Cereb. Cortex N. Y. N.

[bb0045] Chen C.-H., Suckling J., Lennox B.R., Ooi C., Bullmore E.T. (2011). A quantitative meta-analysis of fMRI studies in bipolar disorder. Bipolar Disord..

[bb0050] Costafreda S.G., Brammer M.J., David A.S., Fu C.H.Y. (2008). Predictors of amygdala activation during the processing of emotional stimuli: a meta-analysis of 385 PET and fMRI studies. Brain Res. Rev..

[bb0055] Critchley H.D., Wiens S., Rotshtein P., Öhman A., Dolan R.J. (2004). Neural systems supporting interoceptive awareness. Nat. Neurosci..

[bb0060] Del-Ben C.M., Ferreira C.A.Q., Sanchez T.A., Alves-Neto W.C., Guapo V.G., de Araujo D.B., Graeff F.G. (2012). Effects of diazepam on BOLD activation during the processing of aversive faces. J. Psychopharmacol. Oxf. Engl..

[bb0065] Di Simplicio M., Renner F., Blackwell S.E., Mitchell H., Stratford H.J., Watson P., Myers N., Nobre A.C., Lau-Zhu A., Holmes E.A. (2016). An investigation of mental imagery in bipolar disorder: Exploring “the mind's eye.”. Bipolar Disord..

[bb0070] Endler N., Parker J. (1990). Coping Inventory for Stressful Situations (CISS): Manual.

[bb0075] Etkin A., Egner T., Kalisch R. (2011). Emotional processing in anterior cingulate and medial prefrontal cortex. Trends Cogn. Sci..

[bb0080] Frank D.W., Dewitt M., Hudgens-Haney M., Schaeffer D.J., Ball B.H., Schwarz N.F., Hussein A.A., Smart L.M., Sabatinelli D. (2014). Emotion regulation: quantitative meta-analysis of functional activation and deactivation. Neurosci. Biobehav. Rev..

[bb0085] FSL – FslWiki [WWW Document], n.d. https://fsl.fmrib.ox.ac.uk/fsl/fslwiki. URL https://fsl.fmrib.ox.ac.uk/fsl/fslwiki (accessed 11.9.17).

[bb0090] Godlewska B.R., Browning M., Norbury R., Cowen P.J., Harmer C.J. (2016). Early changes in emotional processing as a marker of clinical response to SSRI treatment in depression. Transl. Psychiatry.

[bb0095] Goldin P.R., Manber-Ball T., Werner K., Heimberg R., Gross J.J. (2009). Neural mechanisms of cognitive reappraisal of negative self-beliefs in social anxiety disorder. Biol. Psychiatry.

[bb0100] Gotlib Ian H., Joormann Jutta, Foland-Ross Lara C. (2014). Understanding Familial Risk for Depression: a 25-Year Perspective. Perspect. Psychol. Sci..

[bb0105] Gottesman I.I., Gould T.D. (2003). The endophenotype concept in psychiatry: etymology and strategic intentions. Am. J. Psychiatry.

[bb0110] Hafeman D.M., Bebko G., Bertocci M.A., Fournier J.C., Bonar L., Perlman S.B., Travis M., Gill M.K., Diwadkar V.A., Sunshine J.L., Holland S.K., Kowatch R.A., Birmaher B., Axelson D., Horwitz S.M., Arnold L.E., Fristad M.A., Frazier T.W., Youngstrom E.A., Findling R.L., Drevets W., Phillips M.L. (2014). Abnormal deactivation of the inferior frontal gyrus during implicit emotion processing in youth with bipolar disorder: attenuated by medication. J. Psychiatr. Res..

[bb0115] Hamilton M. (1967). Development of a rating scale for primary depressive illness. Br. J. Soc. Clin. Psychol..

[bb0120] Hofmann S.G., Sawyer A.T., Fang A., Asnaani A. (2012). Emotion dysregulation model of mood and anxiety disorders. Depress. Anxiety.

[bb0125] Holmes E.A., Geddes J.R., Colom F., Goodwin G.M. (2008). Mental imagery as an emotional amplifier: Application to bipolar disorder. Behav. Res. Ther..

[bb0130] Holmes E.A., Bonsall M.B., Hales S.A., Mitchell H., Renner F., Blackwell S.E., Watson P., Goodwin G.M., Di Simplicio M. (2016). Applications of time-series analysis to mood fluctuations in bipolar disorder to promote treatment innovation: a case series. Transl. Psychiatry.

[bb0135] Ivins A., Di Simplicio M., Close H., Goodwin G.M., Holmes E. (2014). Mental imagery in bipolar affective disorder versus unipolar depression: investigating cognitions at times of “positive” mood. J. Affect. Disord..

[bb0140] Kanske P., Schönfelder S., Forneck J., Wessa M. (2015). Impaired regulation of emotion: neural correlates of reappraisal and distraction in bipolar disorder and unaffected relatives. Transl. Psychiatry.

[bb0145] Knauff M., Kassubek J., Mulack T., Greenlee M.W. (2000). Cortical activation evoked by visual mental imagery as measured by fMRI. Neuroreport.

[bb0150] Kohn N., Eickhoff S.B., Scheller M., Laird A.R., Fox P.T., Habel U. (2014). Neural network of cognitive emotion regulation--an ALE meta-analysis and MACM analysis. NeuroImage.

[bb0155] Lang P.J., Bradley M.M., Cuthbert B.N. (1995). International Affective Picture System (IAPS): Technical Manual and Affective Ratings.

[bb0160] Makino Y., Yokosawa K., Takeda Y., Kumada T. (2004). Visual search and memory search engage extensive overlapping cerebral cortices: an fMRI study. NeuroImage.

[bb0170] Mennin D.S., Holaway R.M., Fresco D.M., Moore M.T., Heimberg R.G. (2007). Delineating components of emotion and its dysregulation in anxiety and mood psychopathology. Behav. Ther..

[bb0175] Mors O., Perto G.P., Mortensen P.B. (2011). The Danish Psychiatric Central Research Register. Scand. J. Public Health.

[bb0180] Nelson H.E., O'Connell A. (1978). Dementia: the estimation of premorbid intelligence levels using the New Adult Reading Test. Cortex J. Devoted Study Nerv. Syst. Behav..

[bb0185] Nolen-Hoeksema S., Wisco B.E., Lyubomirsky S. (2008). Rethinking Rumination. Perspect. Psychol. Sci. J. Assoc. Psychol. Sci..

[bb0190] Ochsner K.N., Silvers J.A., Buhle J.T. (2012). Functional imaging studies of emotion regulation: a synthetic review and evolving model of the cognitive control of emotion. Ann. N. Y. Acad. Sci..

[bb0195] Oldfield R.C. (1971). The assessment and analysis of handedness: the Edinburgh inventory. Neuropsychologia.

[bb0200] Palmer S.M., Crewther S.G., Carey L.M. (2015). A meta-analysis of changes in Brain activity in clinical depression. Front. Hum. Neurosci..

[bb0205] Paus T. (1996). Location and function of the human frontal eye-field: a selective review. Neuropsychologia.

[bb0210] Picard N., Strick P.L. (1996). Motor areas of the medial wall: a review of their location and functional activation. Cereb. Cortex N. Y. N.

[bb0215] Picó-Pérez M., Radua J., Steward T., Menchón J.M., Soriano-Mas C. (2017). Emotion regulation in mood and anxiety disorders: a meta-analysis of fMRI cognitive reappraisal studies. Prog. Neuro-Psychopharmacol. Biol. Psychiatry.

[bb0220] Posner J., Maia T.V., Fair D., Peterson B.S., Sonuga-Barke E.J., Nagel B.J. (2011). The attenuation of dysfunctional emotional processing with stimulant medication: an fMRI study of adolescents with ADHD. Psychiatry Res..

[bb0225] Pruim R.H.R., Mennes M., van Rooij D., Llera A., Buitelaar J.K., Beckmann C.F. (2015). ICA-AROMA: a robust ICA-based strategy for removing motion artifacts from fMRI data. NeuroImage.

[bb0230] Simsek F., Oguz K., Kitis O., Akan S.T., Kempton M.J., Gonul A.S. (2017). Neural activation during cognitive reappraisal in girls at high risk for depression. Prog. Neuro-Psychopharmacol. Biol. Psychiatry.

[bb0235] Skytthe A., Christiansen L., Kyvik K.O., Bødker F.L., Hvidberg L., Petersen I., Nielsen M.M.F., Bingley P., Hjelmborg J., Tan Q., Holm N.V., Vaupel J.W., McGue M., Christensen K. (2013). The Danish Twin Registry: linking surveys, national registers, and biological information. Twin Res. Hum. Genet. Off. J. Int. Soc. Twin Stud..

[bb0240] Smith S.M., Nichols T.E. (2009). Threshold-free cluster enhancement: addressing problems of smoothing, threshold dependence and localisation in cluster inference. NeuroImage.

[bb0245] Spielberger C.D. (1989). State-Trait Anxiety Inventory: Bibliography.

[bb0250] Talairach & Tournoux (1988). Standardized Anatomical Atlas.

[bb0255] Van Reekum C.M., van Reekum C. (2007). Gaze fixations predict brain activation during the voluntary regulation of picture-induced negative affect. NeuroImage.

[bb0260] Wager T.D., Davidson M.L., Hughes B.L., Lindquist M.A., Ochsner K.N. (2008). Prefrontal-subcortical pathways mediating successful emotion regulation. Neuron.

[bb0265] Weissman M.M., Wickramaratne P., Gameroff M.J., Warner V., Pilowsky D., Kohad R.G., Verdeli H., Skipper J., Talati A. (2016). Offspring of depressed parents: 30 years later. Am. J. Psychiatry.

[bb0270] Wiggins J.L. (2017). Neural markers in pediatric bipolar disorder and familial risk for bipolar disorder. J. Am. Acad. Child Adolesc. Psychiatry.

[bb0275] Wing J.K., Babor T., Brugha T., Burke J., Cooper J.E., Giel R., Jablenski A., Regier D., Sartorius N. (1990). SCAN. Schedules for Clinical Assessment in Neuropsychiatry. Arch. Gen. Psychiatry.

[bb0280] Winkler A.M., Ridgway G.R., Webster M.A., Smith S.M., Nichols T.E. (2014). Permutation inference for the general linear model. NeuroImage.

[bb0285] Winkler A.M., Webster M.A., Vidaurre D., Nichols T.E., Smith S.M. (2015). Multi-level block permutation. NeuroImage.

[bb0290] Young R.C., Biggs J.T., Ziegler V.E., Meyer D.A. (1978). A rating scale for mania: reliability, validity and sensitivity. Br. J. Psychiatry J. Ment. Sci..

[bb0295] Zilverstand A., Parvaz M.A., Goldstein R.Z. (2017). Neuroimaging cognitive reappraisal in clinical populations to define neural targets for enhancing emotion regulation. A systematic review. NeuroImage.

