# Supplementary material for: Neural response during emotion regulation in monozygotic twins at high familial risk of affective disorders
Source: Neuroimage Clin. 2018 Nov 13;21:101598. doi: 10.1016/j.nicl.2018.11.008 (PMC6411590; doi:10.1016/j.nicl.2018.11.008)
Supplement: Supplementary file 1 — Supplementary material [file mmc1.docx]

**Supplement to methods**

| **Table S1. Pictures from the International Affective Picture System (IAPS)** | | | | | | | |
| --- | --- | --- | --- | --- | --- | --- | --- |
| Just look | | Maintain | | Reappraise | | Change image | |
| Block | Picture | Block | Picture | Block | Picture | Block | Picture |
| 1 | N7496 | 1 | 9270 | 1 | 2799 | 1 | 6213 |
|  | N5396 |  | 2053 |  | 2141 |  | 6212 |
|  | N7205 |  | 6570 |  | 6560 |  | 9584 |
|  | N7186 |  | 9570 |  | 6571 |  | 6530 |
| 2 | N9210 | 2 | 9911 | 2 | 9800 | 2 | 3051 |
|  | N5870 |  | 2751 |  | 9530 |  | 3160 |
|  | N5830 |  | 5971 |  | 9006 |  | 6242 |
|  | N7550 |  | 9340 |  | 9921 |  | 6250 |
| 3 | N2480 | 3 | 6840 | 3 | 3220 | 3 | 9320 |
|  | N1121 |  | 3180 |  | 9910 |  | 9373 |
|  | N5875 |  | 9470 |  | 9050 |  | 3301 |
|  | N5250 |  | 6020 |  | 9432 |  | 6540 |
| 4 | N7495 | 4 | 2900 | 4 | 3530 | 4 | 6834 |
|  | N6150 |  | 6360 |  | 9181 |  | 9253 |
|  | N5535 |  | 9810 |  | 9582 |  | 1111 |
|  | N7000 |  | 3300 |  | 1930 |  | 9120 |
| 5 | N5740 | 5 | 8230 | 5 | 9561 | 5 | 2750 |
|  | N1670 |  | 9430 |  | 2730 |  | 3100 |
|  | N2485 |  | 9101 |  | 8480 |  | 9500 |
|  | N2575 |  | 3230 |  | 9300 |  | 9592 |
| 6 | N5455 | 6 | 1019 | 6 | 6010 | 6 | 1050 |
|  | N7170 |  | 1300 |  | 3266 |  | 9280 |
|  | N7595 |  | 2661 |  | 6838 |  | 9400 |
|  | N7100 |  | 9611 |  | 6313 |  | 3550 |
| 7 | N2840 | 7 | 9560 | 7 | 9620 | 7 | 2700 |
|  | N5900 |  | 9000 |  | 6022 |  | 3140 |
|  | N5300 |  | 6230 |  | 7380 |  | 6830 |
|  | N2381 |  | 9041 |  | 1201 |  | 9920 |
| 8 | N7217 | 8 | 9560* | 8 | 2691 | 8 | 2753 |
|  | N7025 |  | 9000* |  | 9040 |  | 9571 |
|  | N7224 |  | 6230* |  | 9417 |  | 7361 |
|  | N5660 |  | 9041* |  | 3030 |  | 6510 |
| * The repetition of these four pictures was an error. The last maintain block was therefore excluded from analyses. | | | | | | | |

**Table S1.** Pictures taken from the International affective picture system in each condition and block are denoted with pictures names.

*Masks used in volume of interest analysis*

We made all makes for volume of interest (VOI) analyses with the built-in Harvard-Oxford Cortical and Subcortical Structural atlases in Fslview, part of the FMRIB Software Library (1). The prefrontal cortex (PFC) mask were made by the following command: *fslmaths Superior\ Frontal\ Gyrus.nii.gz -add Middle\ Frontal\ Gyrus.nii.gz -add Inferior\ Frontal\ Gyrus\,\ pars\ triangularis.nii.gz -add Inferior\ Frontal\ Gyrus\,\ pars\ opercularis.nii.gz -add Frontal\ Pole.nii.gz -add Cingulate\ Gyrus\,\ anterior\ division.nii.gz -bin PFC_IAPS_mask.* This mask includes peak activations of aberrant functional magnetic resonance (fMRI) BOLD response in patients with affective disorders vs. control participants during reappraisal within the prefrontal cortex derived from Table 2 in (2).

The mask used for the mental imagery contrast was made with the following command: *fslmaths Angular\ Gyrus.nii.gz -add Cuneal\ Cortex.nii.gz -add Lateral\ Occipital\ Cortex\,\ inferior\ division.nii.gz -add Lateral\ Occipital\ Cortex\,\ superior\ division.nii.gz -add Occipital\ Pole.nii.gz -add Precuneous\ Cortex.nii.gz -add Superior\ Parietal\ Lobule.nii.gz -add Supramarginal\ Gyrus\,\ anterior\ division.nii.gz -add Supramarginal\ Gyrus\,\ posterior\ division.nii.gz -add Lingual\ Gyrus.nii.gz -add Occipital\ Fusiform\ Gyrus.nii.gz -add Intracalcarine\ Cortex.nii.gz -add Supracalcarine\ Cortex.nii.gz -thr 20 -bin imagery_mask.* This mask includes peak activations of fMRI BOLD response during mental imagery versus baseline in healthy individuals according to Table 2 in (3).

Finally, the masks for left and right amygdala were made with the following commands: *fslmaths Left\ Amygdala.nii.gz -thr 20 -bin left_amy_mask* and f*slmaths Right\ Amygdala.nii.gz -thr 20 -bin right_amy_mask*.


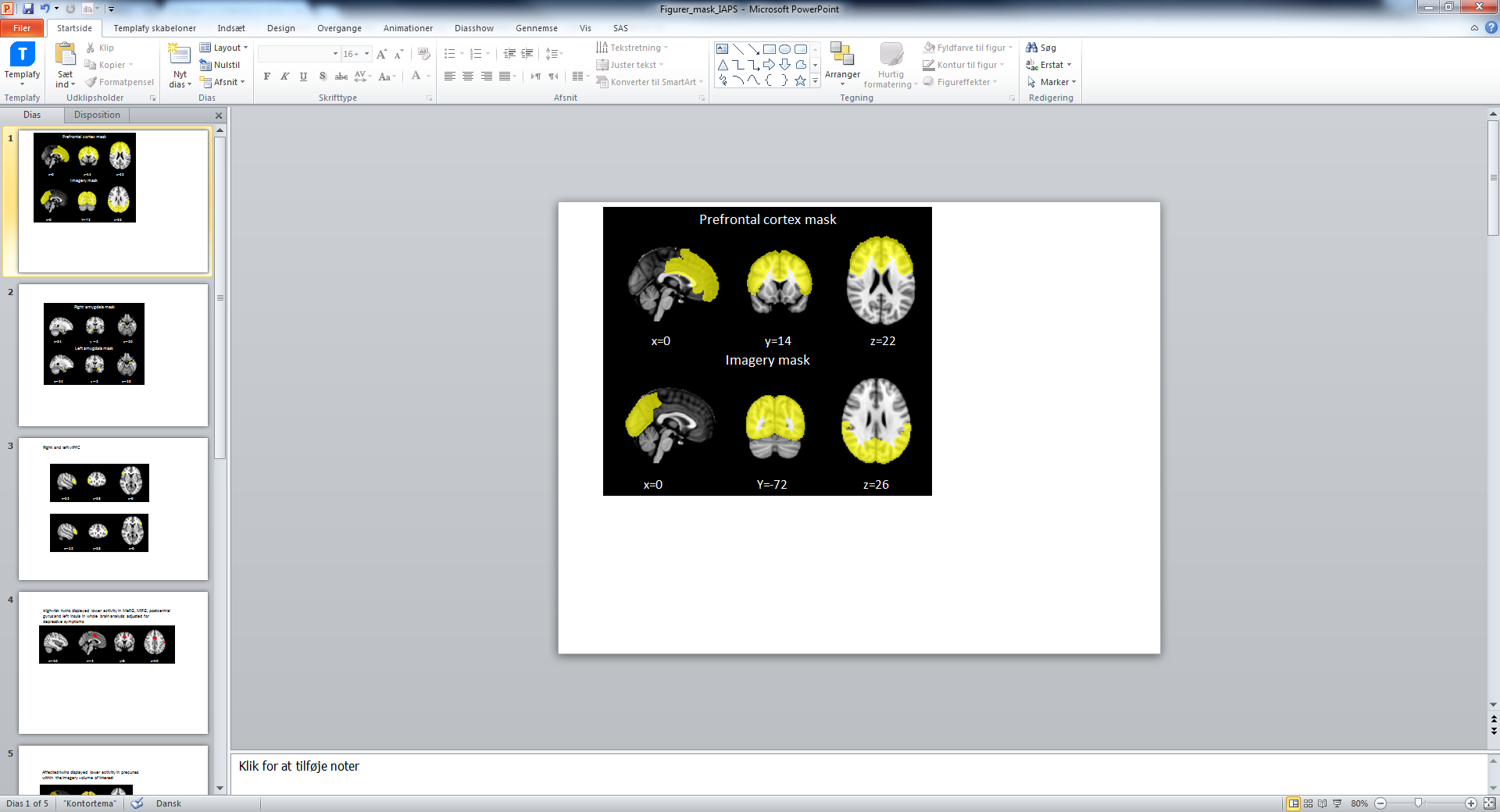

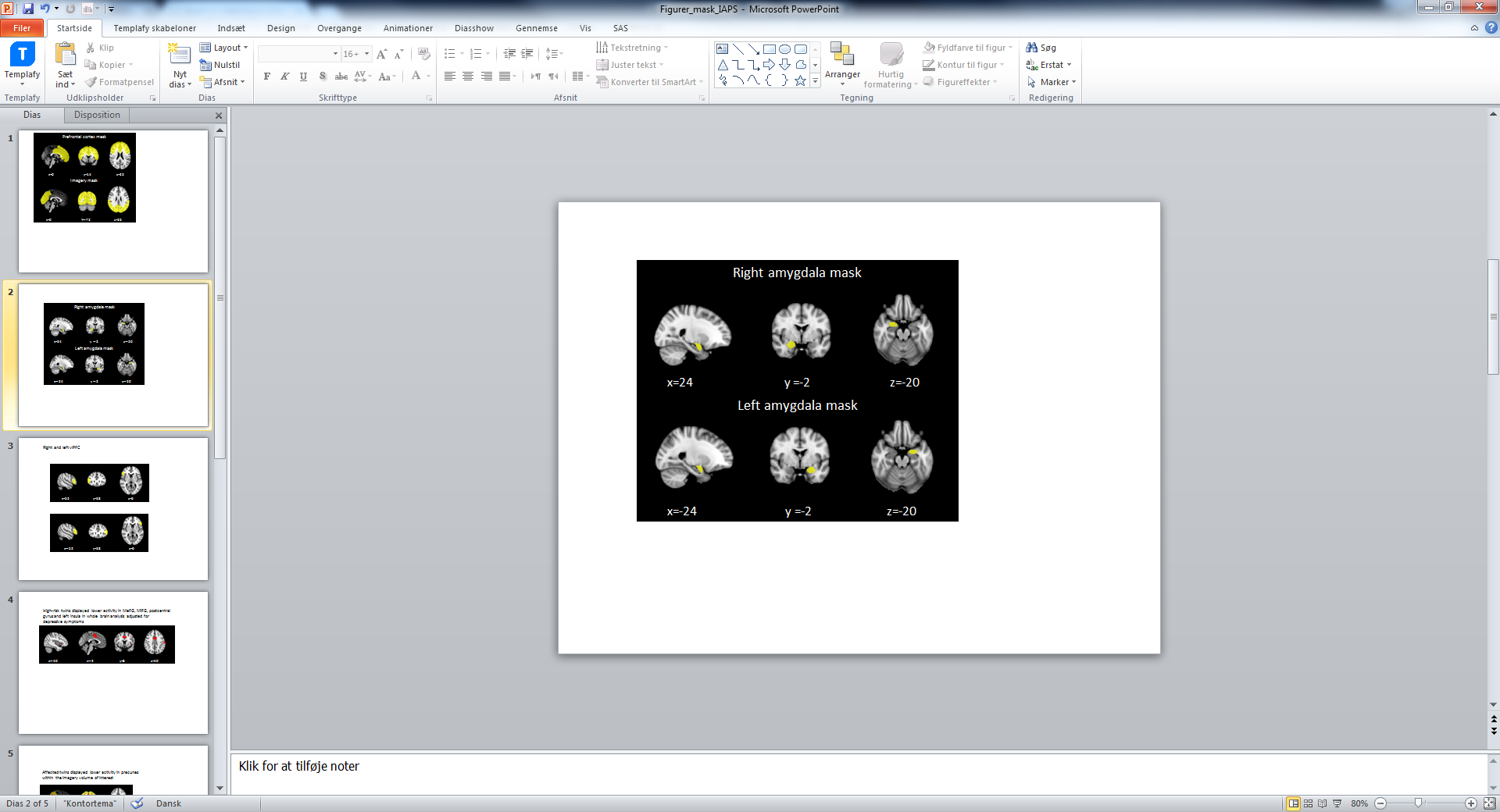


**Supplementary Figure 1.** The mask of prefrontal cortex, part of cortex shown to be involved with mental imagery and left and right amygdala are displayed in yellow superimposed on the Montreal Neurological Institute (MNI) standard brain. Appropriate MNI coordinates were chosen for illustrative purposes.

*Masks used in* [*psychophysiological interaction*](https://www.google.dk/url?sa=t&rct=j&q=&esrc=s&source=web&cd=1&ved=0ahUKEwiM2cDcluTYAhXHaVAKHX6ODIQQFggnMAA&url=https%3A%2F%2Fen.wikipedia.org%2Fwiki%2FPsychophysiological_Interaction&usg=AOvVaw3pj0Aw1R4WVzJmu4HuNBqw) *analysis*

### The masks used to extract time series from seed regions in [psychophysiological interaction](https://www.google.dk/url?sa=t&rct=j&q=&esrc=s&source=web&cd=1&ved=0ahUKEwiM2cDcluTYAhXHaVAKHX6ODIQQFggnMAA&url=https%3A%2F%2Fen.wikipedia.org%2Fwiki%2FPsychophysiological_Interaction&usg=AOvVaw3pj0Aw1R4WVzJmu4HuNBqw) analysis (PPI) functional analysis of right and left ventrolateral prefrontal cortex were made with the following commands: fslmaths Inferior\ Frontal\ Gyrus\,\ pars\ triangularis.nii.gz -thr 20 -bin vlPFC_atlas_mask, followed by the roi option to divide in right and left hemisphere.


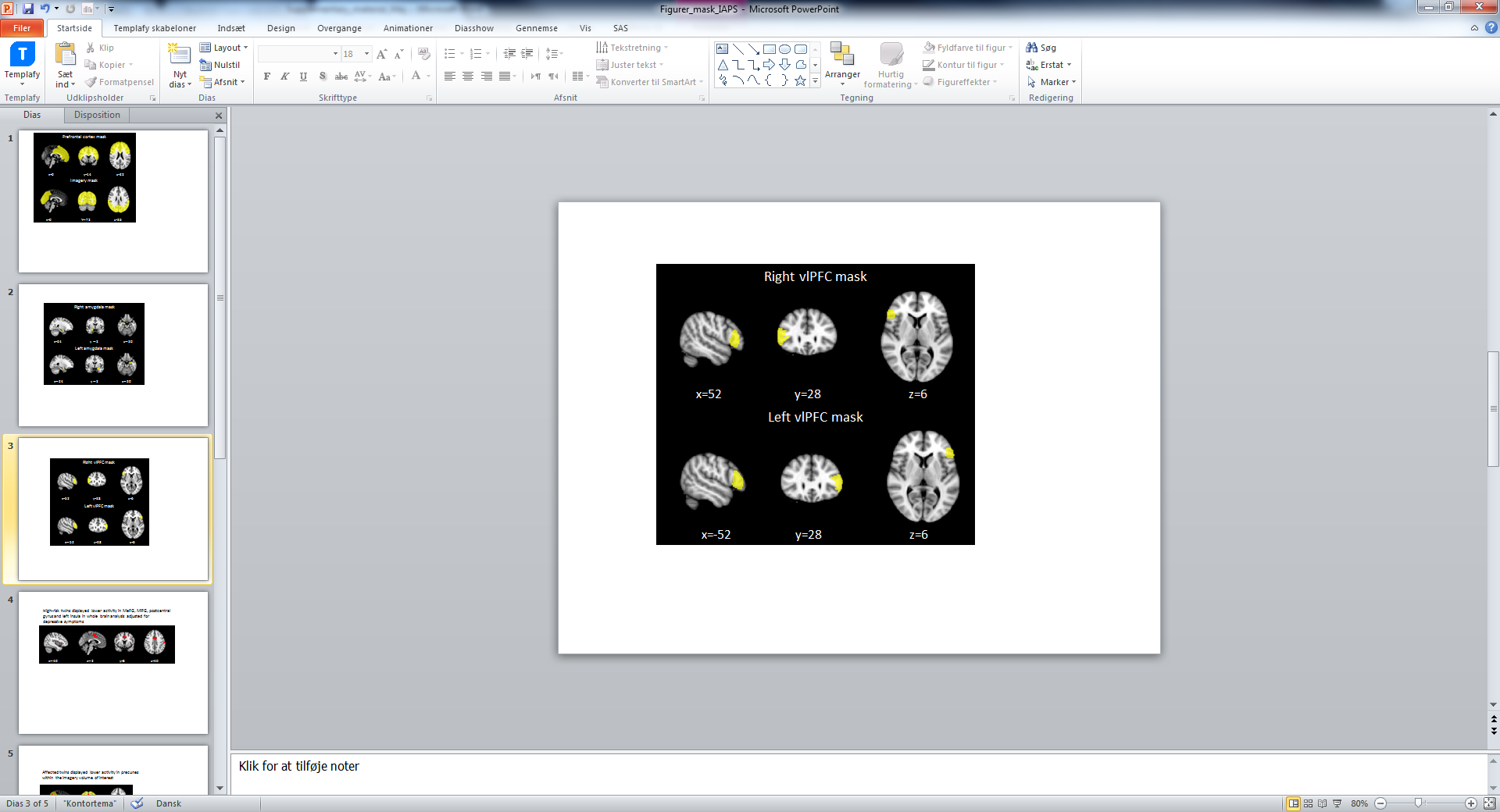


**Supplementary Figure 2.** Masks of left and right ventrolateral prefrontal cortex (vlPFC) are displayed in yellow superimposed on the Montreal Neurological Institute (MNI) standard brain. Appropriate MNI coordinates were chosen for illustrative purposes.

**Supplement to results**


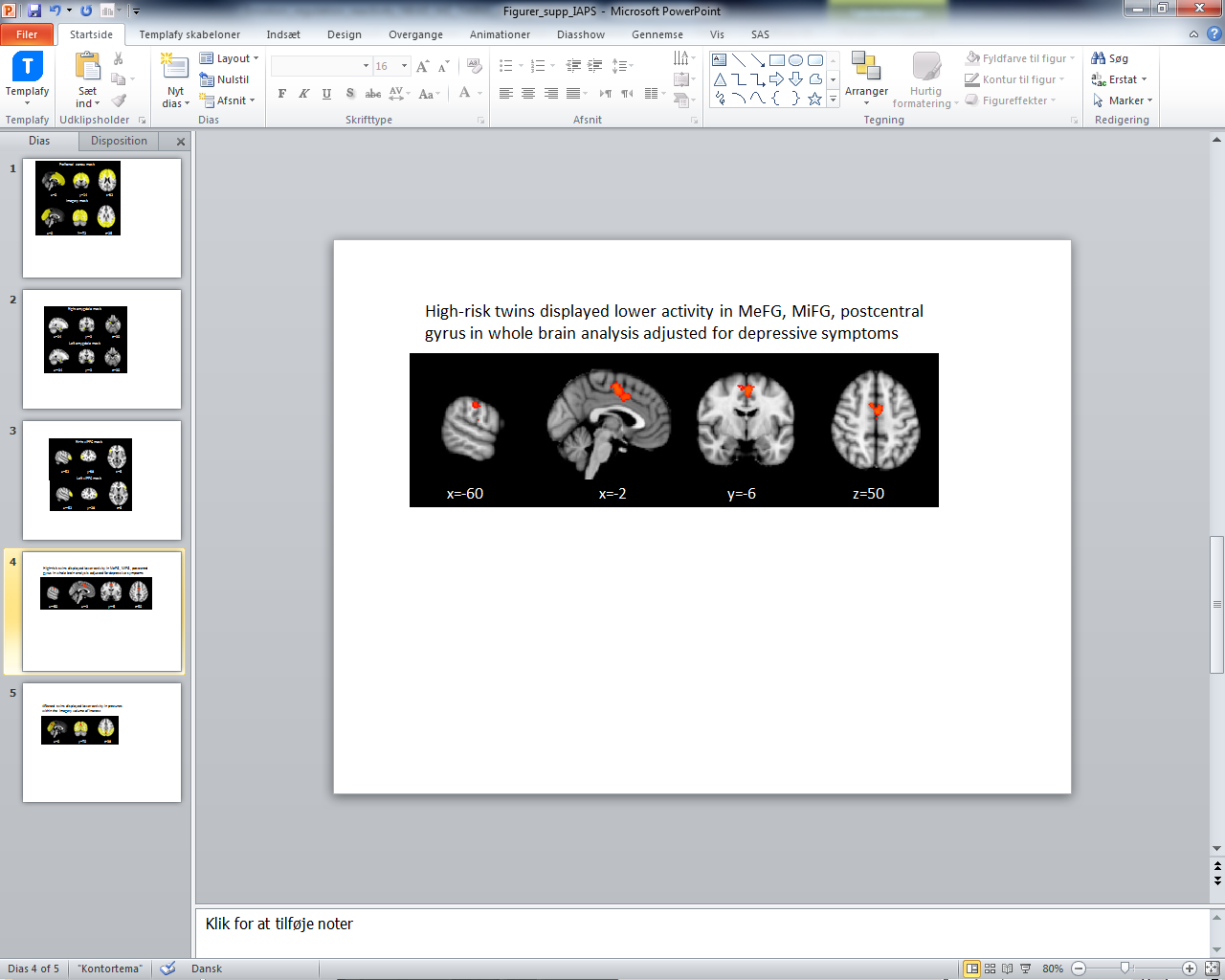


**Supplementary Figure 3.** In whole brain explorative group analysis adjusted for subsyndromal depressive symptoms there was a trend in three-way group analyses that was driven by lower activity in medial frontal gyrus, middle frontal gyrus, postcentral gyrus and left insula in high-risk monozygotic twins compared with low-risk monozygotic twins. Significant clusters are displayed in red superimposed on the Montreal Neurological Institute (MNI) standard brain. Appropriate MNI coordinates were chosen for illustrative purposes.


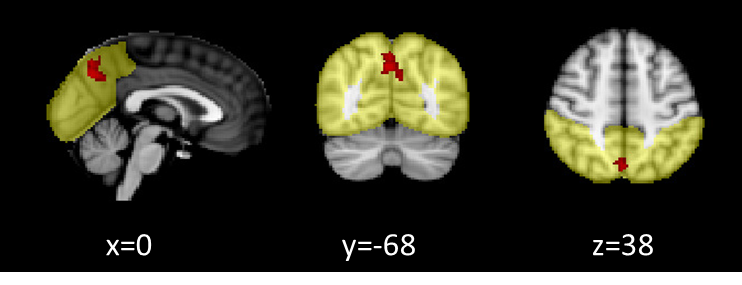


**Supplementary Figure 4.**  In exploratory pairwise group analysis within the imagery volume of interest, affected monozygotic twins displayed a trend towards lower activity in the precunes when compared with low-risk monozygotic twins in the change image > maintain contrast. The trend level cluster is displayed in red and the imagery mask is displayed in yellow superimposed on the Montreal Neurological Institute (MNI) standard brain. The peak cluster MNI coordinates were chosen for illustrative purposes.


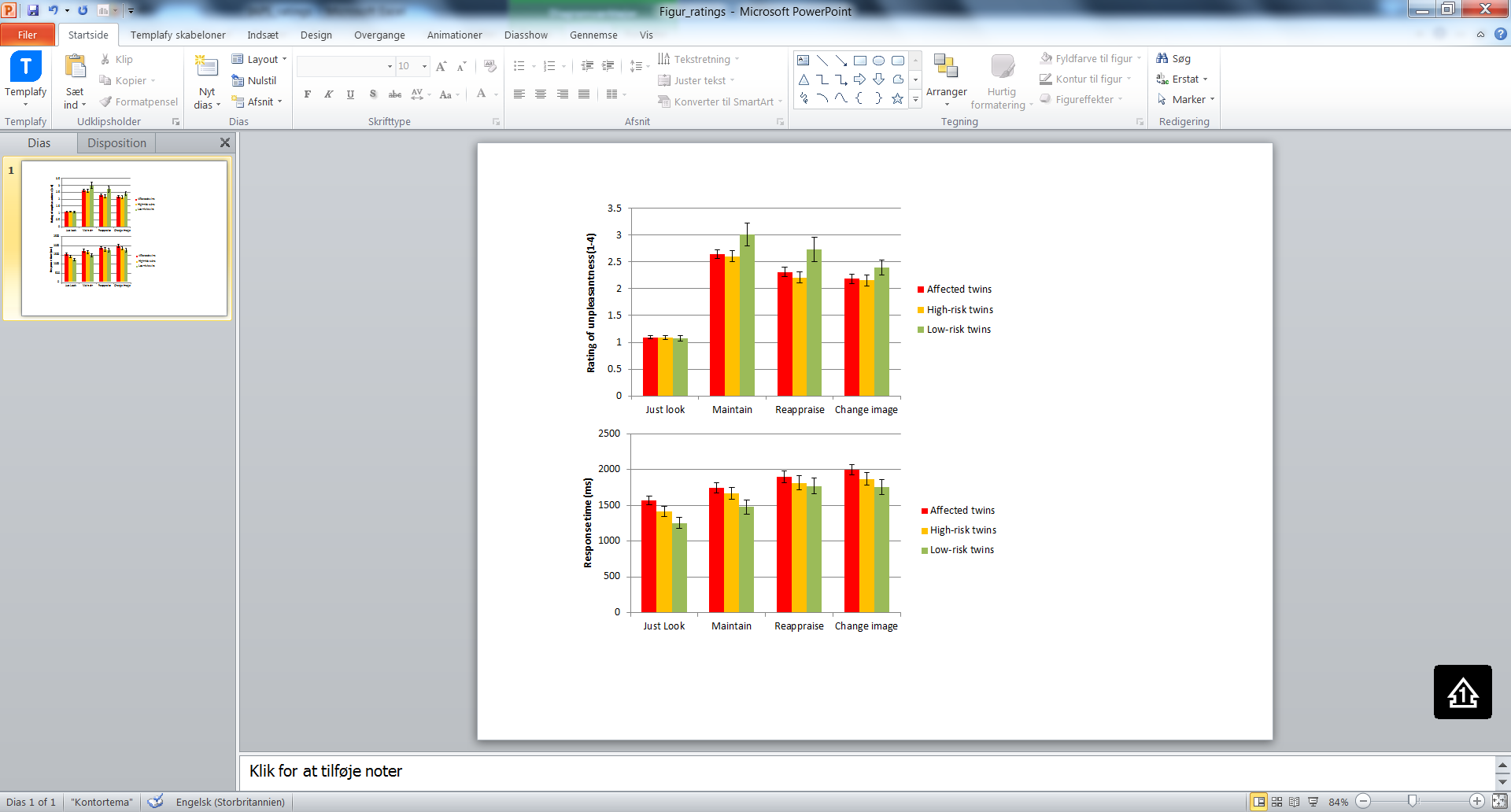


**Supplementary Figure 5.** Ratings and response times of ratings of degree of unpleasantness elicited by neutral or unpleasant pictures form the International Affective Picture System (IAPS) during functional magnetic imaging are displayed as group means for affected, high-risk and low-risk groups. Each of the four conditions ‘just look’ to neutral pictures and ‘maintain’, ‘reappraise’ and ‘change image’ to unpleasant pictures are displayed separately. Group means are computed with mixed models accounting for dependence within twin pairs. Error bars represents the standard error of the mean.


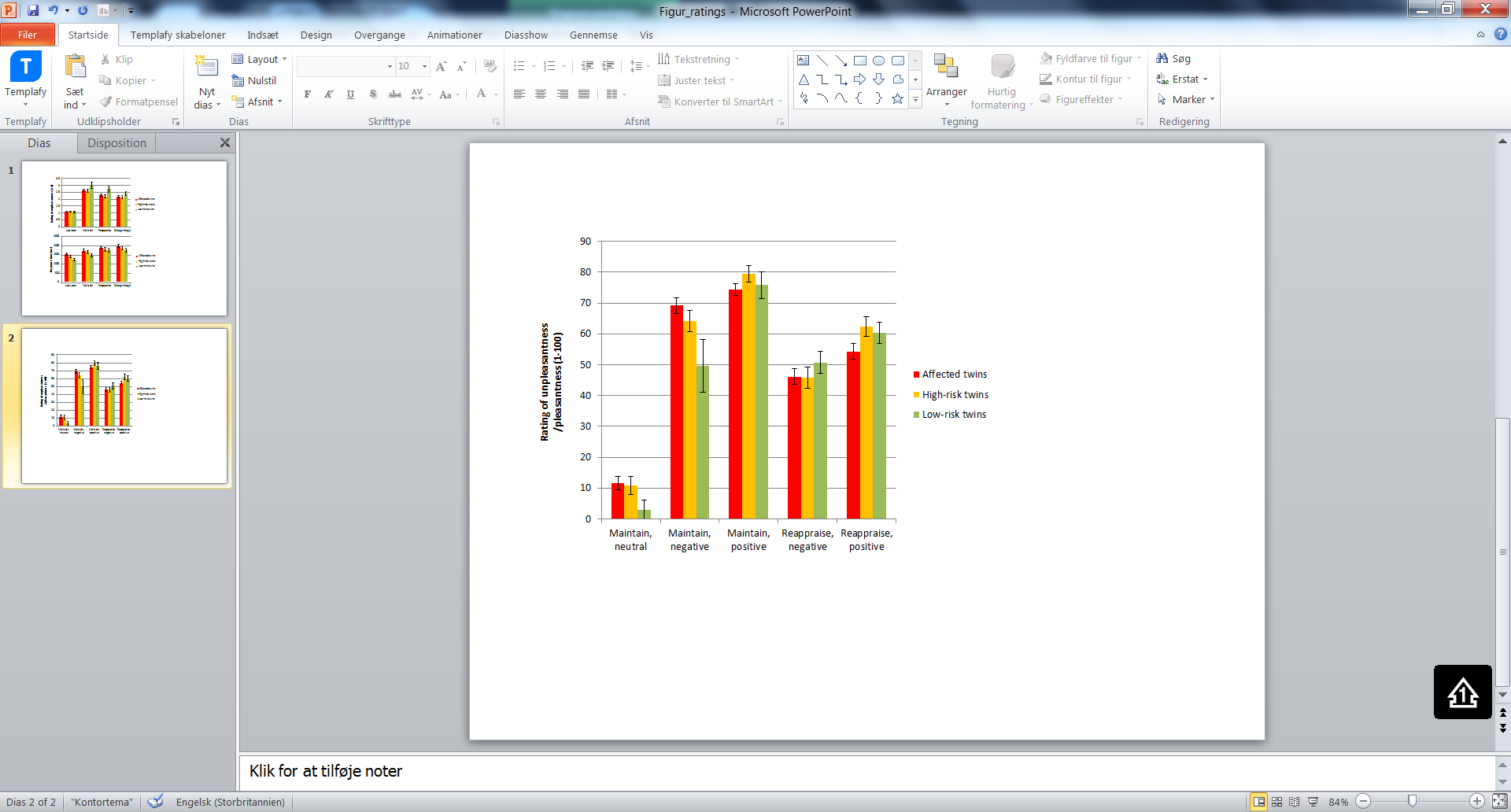


**Supplementary Figure 6.** Ratings of pleasantness or unpleasantness elicited by neutral, positive or negative social scenarios are displayed as group means for affected, high-risk and low-risk groups. Maintain and reappraise conditions are presented separately. Group means are computed with mixed models accounting for dependence within twin pairs. Error bars represents the standard error of the mean.

| **Table S2. Group comparisons of non-medicated affected (*n* = 23), high-risk (*n* = 35) and low-risk (*n* = 28) MZ twins of emotion regulation through reappraisal during fMRI**. | | | | | | | | | | | | | | | | |
| --- | --- | --- | --- | --- | --- | --- | --- | --- | --- | --- | --- | --- | --- | --- | --- | --- |
| Search area | Region | BA | MNI  x y z | | | | | | | | Voxels | | | Peak  p-value | | |
| **Reappraise > maintain** | | | | | | | | | | | | | | | | |
| *High-risk < low-risk* | |  | |  |  | |  | |  | | |  | | |  |  |
| PFC | Medial frontal gyrus | 6 | | -2 | | -6 | | 50 | | 50 | | | .08 | | | |
|  | Medial frontal gyrus | 6 | | 4 | | 8 | | 44 | | 24 | | | .09 | | | |
| PFC_HDRS_^a^ | Medial frontal gyrus | 6 | | -2 | | -6 | | 50 | | 67 | | | .08 | | | |
|  | Medial frontal gyrus | 6 | | 4 | | 8 | | 44 | | 55 | | | .08 | | | |
| a = HDRS refers to adjustment for subsyndromal depressive symptoms | | | | | | | | | | | | | | | |  |
| Abbreviations: VOI = Volume of Interest, FWE =Family Wise Error, BA= Brodmann Area, MNI, = Montreal Neurological Institute, PFC = Prefrontal Cortex, HDRS =Hamilton Depression Rating Scale | | | | | | | | | | | | | | | |  |

**Table S2.** Group comparisons are presented by peak cluster localization in Montreal Neurological Institute (MNI) standard space coordinates and cerebral region with corresponding Brodmann area after conversion to Talairach space, cluster size (number of voxels) and peak P-values. Results are derived from permutation methods allowing modelling the dependence structure within twin pairs. To define clusters, the threshold free cluster enhancement method was used. Trend level results were found by thresholding family-wise corrected images at *P* = .10. Results within the prefrontal cortex volume of interest are presented adjusted and unadjusted for subsyndromal depressive symptoms.
